# Evaluation of an AI Medical Scribe After 236,153 Notes Generated Across Care Levels in a European Health System: Mixed Methods Retrospective Observational Study

**DOI:** 10.2196/90052

**Published:** 2026-07-10

**Authors:** Enni Sanmark, Ville Vartiainen, Johan Sanmark, Katarina Wettin, Lukas Saari, Artin Entezarjou

**Affiliations:** 1 Heart and Lung Center, Helsinki University Hospital, Stenbäckinkatu 8, Helsinki, Uusimaa, 00250, Finland, 358 408446940; 2 Tandem Health, Stockholm, Sweden; 3 Capio Ramsay Santé, Stockholm, Sweden

**Keywords:** artificial intelligence, AI medical scribe, documentation burden, time saving, stress

## Abstract

**Background:**

Clinicians spend a substantial share of their working hours on documentation, contributing to workflow inefficiencies, reduced patient-facing time, and increased burnout. Artificial intelligence (AI) medical scribes have emerged as a promising solution to reduce this burden, yet real-world evidence remains limited and heterogeneous, and data from European health systems are especially scarce. This evaluation combines 2 complementary data sources: objective editing metadata from 236,153 notes generated by 1295 clinicians, describing operational editing behavior within the AI medical scribe, and paired self-reported survey responses from 177 fully onboarded clinicians, capturing perceived change in documentation time and clinician experience.

**Objective:**

This study aimed to evaluate the association of an AI medical scribe on documentation time and clinician experience.

**Methods:**

This observational real-world evaluation was conducted between April 26, 2024, and October 27, 2025, using retrospective paired ratings. The study was carried out across multiple specialties in primary, secondary, and hospital care within Capio Ramsay Santé, a large integrated health care provider operating in Sweden. Eligibility was limited to fully onboarded users, defined as clinicians who had used the scribe for at least 3 months, created more than 100 notes, generated at least 1 document or certificate, and used the conversational edit (“Add or adjust”) feature at least once.

**Results:**

Following the introduction of the AI medical scribe, the estimated time spent on documentation per note was lower than before (4.72 vs 6.69 minutes; −29%, *P*<.001). On a 5-point Likert scale, ratings for the ability to work without stress related to administrative tasks were higher after introduction than before (mean 3.14 vs 2.41; *P*<.001; median change 0 points, 95% CI 0–1), as were ratings for perceived presence with patients (mean 4.33 vs 3.73; *P*<.001; median change 0 points, 95% CI 0–1). The median editing time was 93 seconds, and it did not decrease significantly over continued use.

**Conclusions:**

Among sustained, fully onboarded adopters in a European health care system, use of an AI medical scribe was associated with reductions in self-reported documentation time, administrative stress, and increase of presence with patients, consistent with findings from prior US-based studies. Because the survey cohort represents a highly selected subgroup of users who adopted and continued using the tool mainly in general practice, these associations may not generalize to clinicians who discontinued use or never fully adopted the scribe, and the generalizability across specialties remains unverified. The single-arm observational design and reliance on retrospective self-report are important considerations when interpreting these associations. A limitation of this analysis is that 138,196 notes were excluded because their recorded editing time was 0; these notes may have been used as generated, used as a starting point and later modified in the medical record system, or discarded, which limits the operational interpretation of the editing-time findings.

## Introduction

Integrating artificial intelligence (AI) into health care has created substantial opportunities to not only improve clinical efficiency but also enhance patient care and increase clinicians’ professional satisfaction. One of the most compelling possibilities lies in reducing clinicians’ documentation burden. Although electronic health records (EHRs) have contributed to improved health outcomes [1] and reduced medical errors [2], they have also become a dominant part of clinicians’ workdays and are linked to diminished job satisfaction and increased burnout [3-5]. Research consistently shows that clinicians spend up to half of their working hours on computers, primarily performing documentation tasks [6-9], while less than 30% of time is devoted to direct patient care [10]. In this context, ambient large language model–based AI medical scribes able to transcribe clinician-patient interactions and generate structured draft notes have emerged as a promising and scalable means to substantially reduce documentation load. Thereby, they may be able to free time, improve workflow, support better patient care, improve quality of notes, and ultimately strengthen clinician well-being across specialties and care settings [11-16].

Evidence on the effectiveness of AI-assisted clinical documentation remains limited, heterogeneous, and in part contradictory. Early large-scale deployments, primarily in North America, suggest that AI medical scribes can reduce time spent on documentation, decrease after-hours EHR work, and improve elements of clinician-patient interaction [12-14,16,17]. However, reported benefits vary markedly across clinical specialties, workflows, and implementation strategies, emphasizing the need for context-specific evaluation [12,14]. Conversely, some studies have found no measurable improvements in efficiency, in terms of either time savings or economic outcomes [18]. Complicating matters further, the underlying technology is evolving at a rapid pace, making it challenging to draw stable conclusions about current real-world performance. Despite these uncertainties, health care organizations are making adoption decisions now, and AI medical scribe systems are already in use in nearly all major US health systems [19]. This rapidly evolving landscape underscores the importance of rigorous, context-sensitive evaluation of their effectiveness.

European health systems operate within regulatory and operational conditions that may substantially influence the performance and adoption of AI medical scribe technology [20,21]. Unlike AI medical scribes deployed in the United States, which are generally not classified as medical devices, the tool used in this study is a class I medical device under the EU Medical Device Regulation [21]. This classification imposes specific regulatory obligations that directly shape the product’s design and postmarket behavior.

The AI medical scribe is designed and deployed in compliance with EU Medical Device Regulation and ISO 14971, incorporating formal risk assessment, defined intended use, and documented mitigation strategies that create user-facing safeguards uncommon in unregulated tools. Clinicians cannot transfer AI-generated notes to the medical record without a mandatory review period, and input of large unreviewed text blocks is restricted. Site managers can monitor edit and transfer rates to ensure appropriate use. The system uses natively multilingual large language models for transcription and note structuring, supporting consultations in over 50 languages, including Swedish, without intermediate translation, and monthly audits monitor hallucinations and omissions. Data governance complies with GDPR (General Data Protection Regulation) and national legislation. All processing occurs on EU-based infrastructure, and audio is streamed in real time without persistent storage, ensuring patient privacy.

Combined with Europe’s heterogeneous EHR landscape and multilingual clinical environment, these factors create a context that differs markedly from non-EU settings [22]. Evidence from European countries remains especially limited [23,24], despite their high digital maturity and increasing interest in reducing administrative burden and improving clinician experience through advanced automation.

The aim of this study is to evaluate the association of a fully deployed AI medical scribe on clinical documentation time and clinician experience in primary and secondary care (including hospital care) across multiple specialties. Together, these outcomes provide a comprehensive assessment of the effectiveness and usability of AI-scribe technology in Europe during large-scale implementation in routine clinical practice.

## Methods

### Ethical Considerations

The study does not constitute human participants research, as it involved analysis of deidentified, aggregated survey data for which ethical approval is not required. Clinician survey respondents were informed in writing about the purpose of the study, the voluntary nature of participation, the pseudonymized handling of their responses, and their right to withdraw, and they consented to participation by submitting the survey. No patients were enrolled, contacted, or asked to consent, as no patient-level data were collected or processed explicitly for the purposes of this retrospective study. In routine clinical use, patients are informed at the start of the consultation that an AI-based documentation tool will transcribe the conversation in real time to support note generation, that no audio is stored, and that they may decline without any effect on their care. Verbal consent is obtained before the scribe is activated, and written information about the tool is available on request.

This study analyzed clinician survey responses and operational note metadata; no patient-level data were collected or processed explicitly for the purposes of this retrospective study. Under the Swedish Ethical Review Act (Lag 2003:460 om etikprövning av forskning som avser människor), ethical review board approval is required for research involving the processing of sensitive personal data as defined in Article 9 of the General Data Protection Regulation, or physical interventions on research subjects. As the study involved only professional experience ratings and pseudonymized usage metadata, with no sensitive personal data categories processed, it fell outside the scope of the Act. Survey participation was voluntary, and respondents were informed of the study’s purpose. Capio Ramsay Santé acted as data controller. Capio administered the clinician survey and held the operational usage data from participating units. Metadata exported from the vendor’s system included only the variables required for the preregistered analyses (time stamps, counts, and contextual descriptors). Linkage between survey responses and usage statistics was performed under Capio’s organizational data governance policies. The researchers conducting the final statistical analysis received only pseudonymized, prelinked datasets and had no access to respondent identities at any stage. Pseudonymization was performed before any data were transferred to the analytic environment, and the key was held by the data controller; the analyst (VV) therefore had no opportunity to view identifiable data at any point.

Inclusion and exclusion criteria for both the note-level analysis and the survey cohort were prespecified by the academic coinvestigators (ES and VV) and were not modified by vendor-affiliated authors during analysis. Raw prelinkage data were accessible only to Capio data governance staff and to vendor engineers under contractual obligations; the analytic dataset shared with the research team was pseudonymized and prelinked. All statistical analyses were performed by VV, who has no vendor affiliation and received no compensation for this work.

### Participants, Setting, and Exposure

This observational study was conducted between April 26, 2024, and October 27, 2025, in health care facilities run by Capio—part of Ramsay Santé. The study uses 2 data sources. First, metadata was gathered from all participating units which implemented the same Conformité Européenne–marked AI medical scribe (Tandem AI Medical Scribe, version 1.0‐1.1; Tandem Health AB). Second, a survey was conducted among fully onboarded professionals to gather subjective assessments on the use of the scribe. Survey responses were linked to exported metadata on secure infrastructure under organizational data governance policies. Metadata export from the vendor’s system included only the elements required for the preregistered analyses (time stamps, counts, and contextual descriptors) and did not contain any personal data of the participants.

The study adhered to the SQUIRE (Standards for Quality Improvement Reporting Excellence) guideline and was preregistered on the Open Science Framework on October 7, 2025. The preregistration was filed on October 7, 2025, approximately 3 weeks before the closing of the data collection window (October 27, 2025), and is therefore effectively retrospective with respect to the note-level metadata, which was continuously accumulating from April 2024 onward. The survey component, however, which is the source of the primary outcome (paired recall of documentation time and the Likert ratings), was dispatched to participants after the preregistration date and was therefore prospectively registered. Three additional analyses were added to the original preregistered plan: a linear mixed-effects model, regression models examining predictors of perceived change, and an inverse-probability-weighted (IPW) nonresponse sensitivity analysis. These were incorporated to strengthen the quality and interpretability of the findings and should be considered exploratory rather than confirmatory. No descriptive or inferential analyses of the accumulated metadata had been inspected by the analyst prior to drafting these additions; only the predefined primary and secondary outcomes had been examined.

Primary outcome was defined as change in self-reported time used to complete a typical clinical note with and without the scribe. Self-reported paired recall was chosen as the primary outcome because it was the only metric for which a comparative preimplementation baseline could be constructed: objective within-tool editing time captures only activity inside the AI medical scribe interface (ie, the time the clinician spent editing the draft note within the Tandem application before transferring it to the EHR) and cannot represent the full documentation workflow clinicians performed before its introduction (eg, free-text typing, EHR navigation, and dictation). Objective measurement of absolute time spent on documentation is notoriously difficult in health care settings, where clinicians’ work is highly fragmented and frequently interrupted, and prior studies have reported substantial limitations and methodological failures in attempts to capture it directly [16]. Paired recall, although subject to well-documented overestimation of absolute time, correlates moderately with observed time and is widely used as a pragmatic surrogate in large-scale clinician workflow research [25-27]. We acknowledge that this places a subjective measure at the center of the efficiency analysis, a limitation discussed further in the Discussion section, and therefore reports objective editing time alongside the primary outcome as a complementary operational metric. Secondary outcomes were change in responses to questions “I can work without feeling stressed by administrative tasks” and “I feel fully present with the patient during the consultation” in a 5-point Likert scale. Additional outcomes were yes/no responses to questions related to user satisfaction–related statements.

The exposure consisted of routine use of the AI medical scribe within the health care provider’s clinical operations following standard implementation. The AI medical scribe used in the study generates a draft patient record note based on the clinical encounter, which the user can edit, by typing, dictating, or by providing natural language verbal instructions, before final approval. The workflow is essentially the same in both the desktop and mobile interfaces. Deployment included a structured onboarding program delivered by clinician-informed customer success staff from the vendor: an initial 2-hour foundational session covering system introduction, first note generation, and transfer of the note to the EHR, followed by two 1-hour reinforcement sessions at approximately 4 and 8 weeks to review advanced features and address local workflow needs.

Additionally, the health care provider held a 1-hour session with management and senior clinicians focusing on responsible change management, as well as a separate 1-hour course for all end users focusing on how to use the system responsibly and safely with patients in clinical practice. Adoption at each site was then supported by designated superusers, while the centralized IT organization ensured installation, integration, and basic operability across all participating clinics.

### Survey

The survey was sent to all fully onboarded clinicians by email with 2 additional reminders when necessary. The inclusion criteria were (1) using the scribe for ≥3 months, (2) creating >100 notes with the scribe, (3) generating at least 1 document or certificate, and (4) using the conversational edit (“Add or adjust”) feature at least once. The survey was developed specifically for this evaluation, with some clinician Likert items adapted from a patient survey by Mishra et al [28]. The complete survey is presented in Multimedia Appendix 1.

Time was assessed as the self-reported estimated number of minutes required to complete a typical clinical note with and without the scribe. The survey also included paired 5-point Likert scale items to measure perceived administrative burden (“I can work without feeling stressed by administrative tasks”) and clinician presence (“I feel fully present with the patient during the consultation”) with and without the scribe. Additional survey items were yes/no responses to questions related to user satisfaction–related statements “Tandem is easy to use,” “I want to continue using Tandem,” and “Would you recommend Tandem to colleagues in your or other organizations?”

### Note Metadata

Each note contained pseudonymized linking to the professional for whom the note was generated as well as the clinical site. Recorded edit time per note was estimated from the automatically generated metadata and was defined as the difference between the first and the last time stamps (YYYY-MM-DD HH:MM:SS.mmm) at which the user inputted a keyboard edit or used dictation or conversational edit mode to insert or remove any character from the generated note.

Collected variables and definitions are specified in Textbox 1.

Textbox 1. **Variables and definitions.**

- Profession: Each note is associated with a unique clinician user account. Clinicians access the artificial intelligence medical scribe using strong electronic identification (a health care professional smart card or a government-issued electronic ID application linked to their national personal identity number), which ensures that each account corresponds to a specific licensed individual. For each note, the clinician’s profession was taken from the account-level role metadata and treated as the note-level profession variable. Notes generated by users with a tagged profession of “clinic admin” or “medical secretary” were considered nonclinical activity and were excluded from the analytic cohort.
- Visit type: The visit type was derived from the template used when generating the note. Clinicians actively choose a note template at the start of documentation, and templates are designed and maintained by the health care provider and vendor’s operations team, who know for which clinical context each template is intended. Each distinct template name was thus mapped to 1 of 4 encounter categories: physical (in-person visits and procedures), digital (remote written or online contacts), telephone, or admin or other, with unmatched templates falling into the admin or other category.
- Platform: Clinicians could log in either via a native desktop application or via a web browser on their desktop computer, or on their mobile devices. The platform could thus capture what access method was associated with each note, enabling categorization to desktop or mobile access.

### Statistical Analysis

All statistical analyses were performed in R (version 4.3.3; R Foundation for Statistical Computing). Two complementary datasets were analyzed: (1) note-level editing metadata and (2) clinician-level survey responses describing use of the AI-assisted documentation system. Editing time was defined as the interval between the first and last recorded input event (keyboard edit, dictation, or conversational edit) on a given note, without exclusion of idle time; that is, periods of inactivity within an open editing session are included in the measured duration. This definition captures only activity within the AI medical scribe interface. In contrast, clinician-reported documentation time was intended to reflect all tasks performed within the EHR for a single patient encounter, including activities outside the AI medical scribe interface.

Editing durations displayed a pronounced right-skew with extreme values attributable to system artifacts (eg, notes left open for prolonged periods). To mitigate undue influence of these outliers, we applied a 3-hour cutoff, excluding notes with edit durations >10,800 seconds. We also excluded notes with no recorded edits from the primary analysis. As a sensitivity analysis, notes without recorded edits were assigned an editing time of zero seconds to evaluate the potential impact of excluding these notes. Edit times were further log-transformed to stabilize variance and reduce skewness. To examine within-user changes in editing time, we compared each user’s first 2 months of recorded note activity with the remainder of the study period.

To evaluate factors associated with editing duration at the note level, we fit a linear mixed-effects model with log-transformed edit time as the dependent variable. Fixed effects included visit type, profession, clinic type, and interaction platform. Clinic type was operationalized as a categorical variable distinguishing primary care from specialist outpatient units within the Capio network.

A random intercept for clinician was included to account for within-clinician correlation arising from repeated notes. Model assumptions were assessed visually via Q-Q plots of residuals and residual-versus-fitted plots. This graphical evaluation indicated modest deviations from normality in the tails, which is expected with large-scale operational data but did not materially affect inference.

Since Likert-type responses and recall measures were not normally distributed, paired Wilcoxon signed-rank tests were used to compare editing time recall (before vs after), perceived stress (without vs with AI medical scribe), and perceived presence (without vs with AI assistance). To examine whether objective editing behavior was associated with subjective experience, we fitted clinician-level linear regression models with change in perceived stress, change in perceived presence, or change in perceived documentation time as the outcome. The objective editing time predictor was the clinician-level median editing time across edited notes after artifact filtering, expressed in seconds. Median rather than mean editing time was used to reduce the influence of extreme editing durations. Perceived ease of use (“Tandem is easy to use,” yes/no) was included as a binary predictor. Although note-level edit times were log-transformed in the mixed-effects model because of their right-skewed distribution, the clinician-level regression models used raw clinician-level median seconds to retain direct interpretability. Change scores were calculated as the paired within-clinician difference between the postimplementation rating and the recalled preimplementation rating, so the regression coefficient should be interpreted as the change in the outcome per unit change in the predictor at the clinician level. In addition to objective editing time, perceived ease of use (“Tandem is easy to use,” yes/no) was included as a binary predictor in the regression models examining associations with self-reported outcomes. Because the regression analyses modeled clinician-level change scores, these variables were treated as approximately continuous and analyzed using linear regression. Also, as survey outcomes were measured at the clinician level, note-level editing metrics were aggregated to clinician-level summaries prior to analysis. For each clinician, we calculated the median editing time across all notes generated during the observation period after artifact filtering. These clinician-level editing time summaries were then linked to survey responses using the pseudonymized clinician identifier. Regression analyses therefore examined associations between clinician-level editing behavior and clinician-level changes in perceived outcomes. For completeness, sensitivity analyses using median edit times instead of mean log-edit times produced consistent conclusions. For yes/no survey items, the proportion answering “yes” was calculated, and 95% CIs were obtained using the exact binomial method.

To assess the potential impact of survey nonresponse bias, inverse probability weighting was applied in sensitivity analyses. A logistic regression model estimated the probability of survey response using clinician-level metadata variables (profession, clinic type, note volume, editing time, and implementation timing). Stabilized weights derived from the predicted probabilities were used in weighted linear regression models examining associations between objective editing time, perceived ease of use, and clinician-reported outcomes. Extreme weights were truncated at the 1st and 99th percentiles.

## Results

### Overview

**Figure 1.**
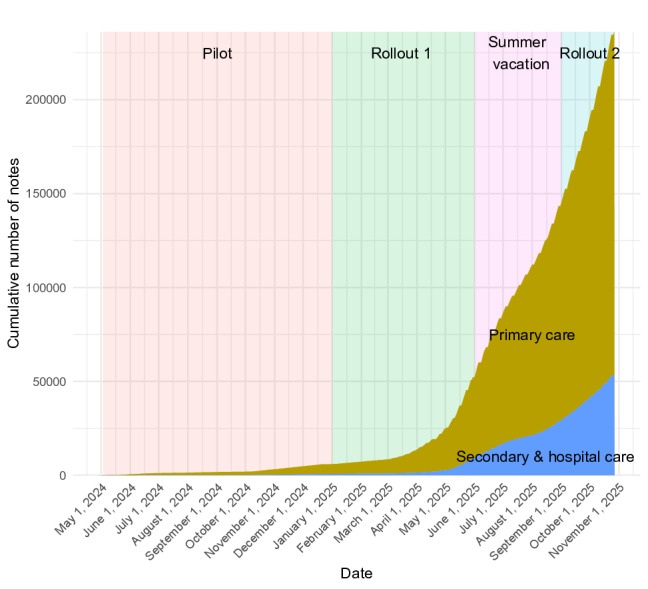
Cumulative number of notes generated during different stages of implementation in primary and secondary care (including hospital care).

The original dataset contained metadata from 376,596 clinical notes. The most active user had generated 2474 clinical notes. After the filtering and removal of artifacts the final dataset contained 236,153 unique notes across 1295 participants. We excluded 138,196 notes with no recorded edits and 2247 notes with editing times exceeding 3 hours. The exclusion of unedited notes was prespecified to ensure that the primary efficiency outcome reflected active documentation work rather than draft generation events that may or may not have entered the clinical record. Although the system tracks note-level transfer events to the EHR for operational monitoring by site managers, these transfer status data were not made available to the research team at the individual note level. This reflected a scope-of-extraction decision at the time of analysis rather than a contractual or GDPR-related restriction, and the linkage of transfer status to editing metadata is technically recoverable in future work. Furthermore, EHR log data were not accessible to the researchers. As a result, it could not be determined with certainty whether unedited notes were accepted as generated, discarded, or modified within the EHR after transfer. The cumulative number of notes and implementation periods are presented in Figure 1.

The 3 most common professions were general practitioners (861/1295, 66.5%), physiotherapists (79/1295, 6.1%), and orthopedic surgeons (63/1295, 4.9%). The remaining 22.5% (292/1295) of participants represented a wide variety of professions including various medical specialties, nurses, dentists, and optometrists (Multimedia Appendix 2). A total of 1302 clinicians had generated at least 1 note in the system; after exclusion of 7 nonclinical accounts (clinic admin and medical secretary), the analytic cohort comprised 1295 clinicians, of which 517 met survey inclusion criteria, and of which 451 were surveyed. Of the 451 surveyed, 177 (39.2% response rate among those invited; 34.2% of the 517 eligible users) clinicians responded, who together created 67,679 notes. Due to missing data, 322 nonresponders and 152 responders were available for the IPW analysis (Multimedia Appendix 3). The survey was distributed to a purposive sample covering the organization’s main clinical settings (primary care, secondary outpatient care, and hospital-based care), while the 66 clinicians outside this sampling frame worked in smaller specialist units. This scope applies only to the survey component, as the objective usage metrics were derived from the full eligible user population. A comparison between notes generated by responders and nonresponders is shown in Multimedia Appendix 4.

### Metadata-Related Results

**Figure 2.**
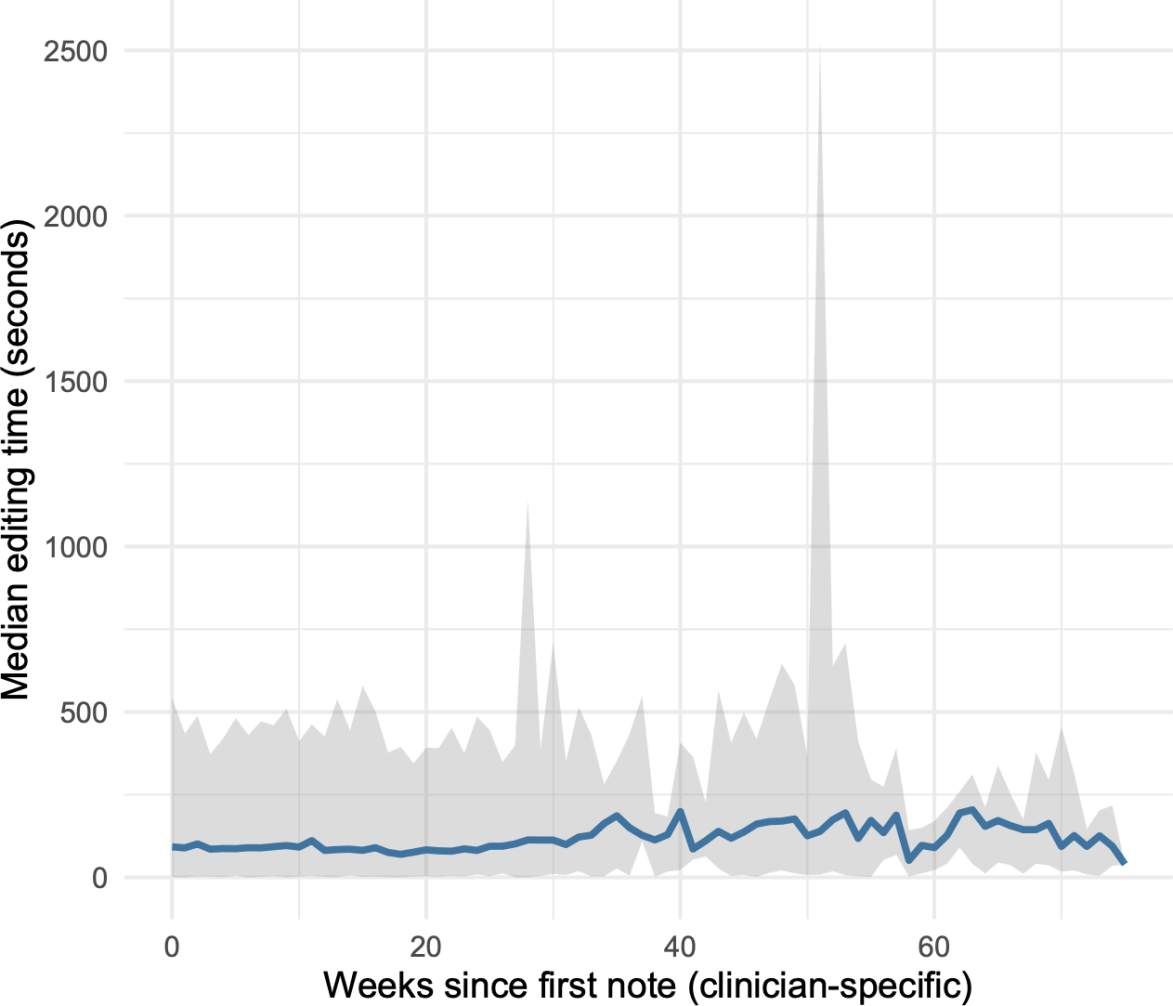
Median editing time as a function of time. The time is defined as weeks from the onboarding for each user separately. The curve shows the weekly medians across clinicians, with the shaded area indicating variability (2.5th-97.5th percentiles).

The median editing time was 93 seconds, whereas mean editing time was 226 seconds after applying the designated artifact cutoff of 3 hours, indicating a highly skewed distribution (Multimedia Appendix 5). The median remained stable in the sensitivity analysis: in the full dataset of generated notes, the median was 94 seconds, and with a 30-minute cutoff, the median was 87 seconds. When notes were stratified into those generated within the first 2 months of use versus those generated thereafter, the median editing times were 84.0 seconds and 84.5 seconds, respectively, suggesting that no substantial learning curve was observed (Figure 2). In a sensitivity analysis including notes without recorded edits as zero-second editing events, the overall median editing time decreased substantially but the comparison between early and later usage periods remained similar (20 seconds vs 22 seconds). The proportion of notes without recorded edits was also comparable between periods (42.4% vs 41.2%).

The effects of visit type and platform on editing time were studied using a linear mixed-effects model, as presented in Table 1. Differences between professions and clinic types were minimal and statistically nonsignificant and were therefore excluded from the final model. Descriptive analysis comparing characteristics of notes with versus without edits is presented in Multimedia Appendix 6.

**Table 1.** Linear mixed-effects model for log transformed edit time.

Variable	β [(e^β^−1) ·100%]	*P* value	95% CI (relative time difference CI)^a^
Visit type			
Physical	Reference	N/A^b^	N/A
Digital	−0.9599 (−61.7%)	<.001	−1.080 to −0.840 (−66% to 57%)
Telephone	−0.7988 (−55.0%)	<.001	−0.872 to −0.726 (−58% to 52%)
Admin/other	−1.060 (−65.3%)	<.001	−1.097 to −1.024 (−67% to 64%)
Platform^c^			
Desktop	Reference	N/A	N/A
Mobile	0.7010 (+102%)	<.001	0.580 to 0.822 (79% to 128%)

a [(eβ-1)·100 %] gives relative time difference to reference.

b N/A: not applicable.

c Platform data were unavailable for 7.7% of cases; detailed information is provided in Multimedia Appendix 6.

### Survey-Related Results

**Figure 3.**
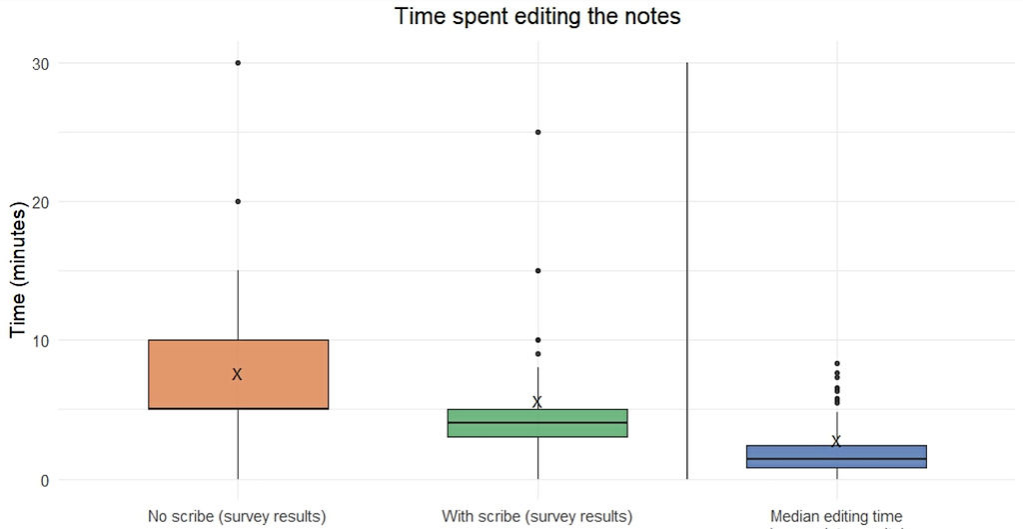
Self-estimated time to complete a single clinical note with and without the artificial intelligence (AI) medical scribe, alongside the objectively measured median editing time for each clinician. The 2 metrics capture different scopes of work: self-reported documentation time is intended to reflect all tasks the clinician associates with completing a note in the electronic health record for a single encounter (including activities outside the AI medical scribe interface, such as navigation, prescriptions, and referrals), whereas objectively measured editing time reflects only activity within the AI medical scribe workflow between the first and last recorded input event. X denotes the mean. Orange indicates no scribe, green indicates with scribe, and blue indicates median editing time.

**Figure 4.**
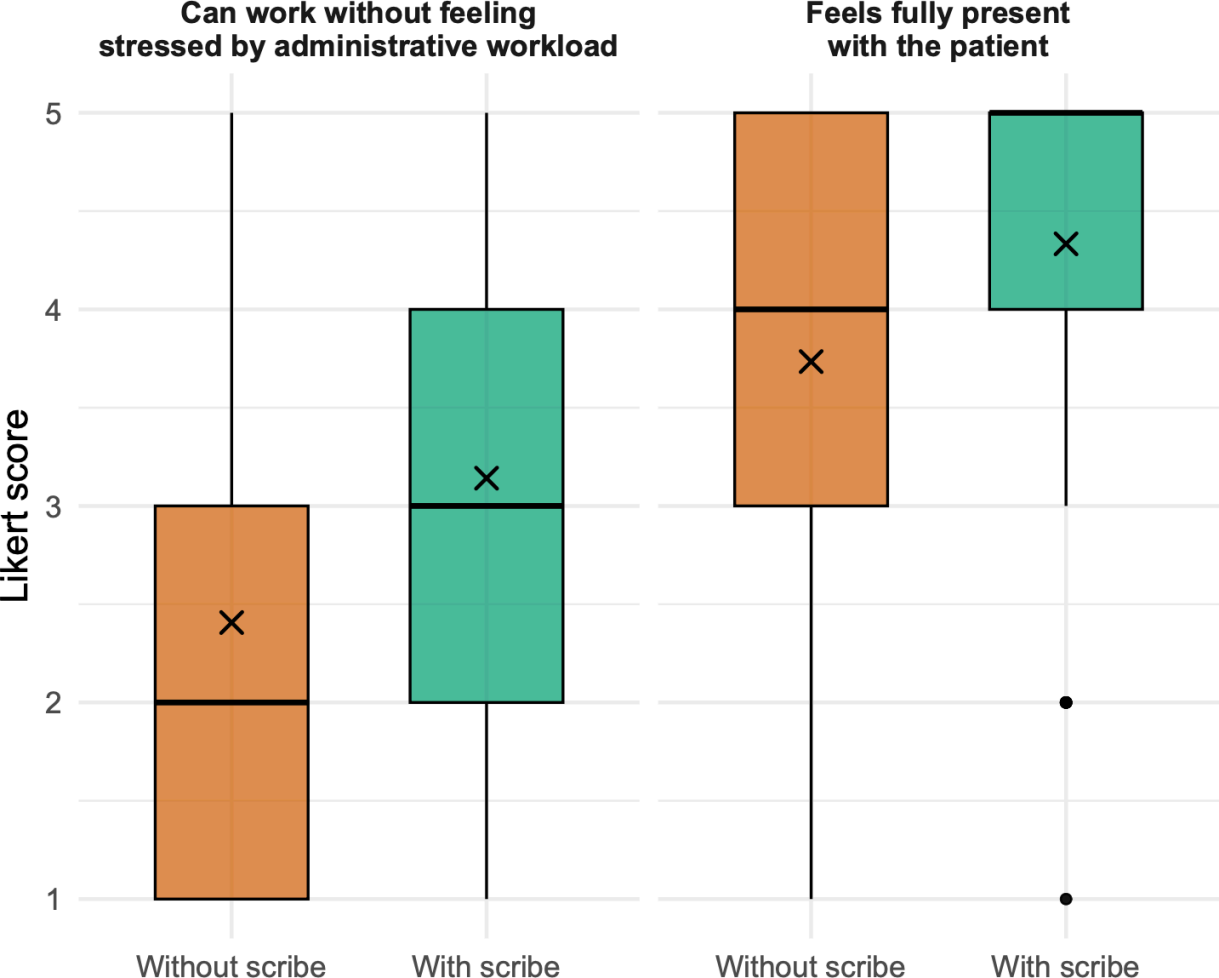
Likert scale answers to questions assessing ability to work without stress from administrative tasks and feel fully present with the patient with and without the artificial intelligence medical scribe. X denotes the mean of the answers. Orange indicates without scribe and green indicates with scribe.

In the predefined primary outcome, estimated time spent on documentation was lower after the introduction of the AI medical scribe than before (6.69 vs 4.72 minutes, *P*<.001; Figure 3), corresponding to a paired decrease of a median of 2 minutes per note (95% CI 1‐2 minutes). On a 5-point Likert scale (Figure 4), mean ability to work without stress related to administrative tasks was higher after the introduction than before (2.41 vs 3.14; *P*<.001), although the median change was 0 points (95% CI 0‐1). Similarly, mean perceived presence with patients was higher after the introduction than before (3.73 vs 4.33; *P*<.001), with a median change of 0 points (95% CI 0‐1). Because the median change was 0 for both Likert outcomes, the statistically significant shifts reflect a consistent directional shift among a subset of respondents rather than a uniform change across the cohort; the Wilcoxon signed-rank test detects such shifts even when the median paired difference is zero. The Likert shift distributions are shown in Multimedia Appendix 5.

In the survey items indicating yes/no answer, 80.2% (142/177) (95% CI 73.6%‐86.0%) said that the scribe was an improvement for their profession, 91.0% (161/177) (95% CI 86.1%‐95.1%) found it easy to use, 91.5% (162/177) (95% CI 86.1%‐95.1%) wished to continue using the scribe, and 84.2% (149/177) (95% CI 78.1%‐89.6%) would recommend colleagues at other clinics to start using the scribe.

Three linear regression models examined whether objectively measured clinician-level median note-editing time and perceived ease of use predicted self-reported changes in documentation time, administrative stress, and patient presence. Lower objective editing time was significantly associated with reduced administrative stress (β=−0.003*,* 95% CI −0.005 to −0.001; *P*=.01), but it did not significantly predict perceived documentation time (β=0.006, 95% CI 0.000-0.012; *P*=.06) or presence with patients (β=−0.001, 95% CI −0.002 to 0.001; *P*=.52).

In contrast, perceived ease of use was a significant predictor in all models: higher ease-of-use ratings were associated with reduced administrative stress (β=1.796, 95% CI 1.043-2.549; *P*<.001), increased patient presence (β=1.199, 95% CI 0.578-1.821; *P*<.001), and lower perceived documentation time (β=−4.144, 95% CI −6.156 to −2.132; *P*<.001). The magnitude of the ease-of-use coefficient for perceived documentation time (β≈−4.1) reflects the wide scale of the recall-time outcome (minutes per note) rather than an unusually large standardized effect. These ease-of-use estimates should be interpreted with caution: 91.5% (162/177) of the respondents rated the scribe as easy to use, so the reference group consisted of only a small subgroup (approximately 15 respondents). Results from IPW models were similar to those from the primary analyses, suggesting that nonresponse bias did not materially affect the observed associations (Table 2).

**Table 2.** Linear regression models (unweighted vs IPW^a^-weighted) for changes in ability to work without stress of administrative tasks, presence with patients, and perceived time used for documentation^b^*.*

Outcome	Predictor	Unweighted β (95% CI)	IPW^a^-weighted β (95% CI)	Unweighted *P* value	IPW-weighted *P* value
Δ Stress	Median edit time (seconds)	−0.003 (−0.005 to −0.001)	−0.003 (−0.006 to −0.001)	.01	.01
	Perceived ease of use (yes vs no)^c^	1.796 (1.043 to 2.549)	1.62 (0.85 to 2.389)	<.001	<.001
Δ Presence	Median edit time (seconds)	−0.001 (−0.002 to 0.001)	0 (−0.002 to 0.002)	.52	.69
	Perceived ease of use (yes vs no)^c^	1.199 (0.578 to 1.821)	0.971 (0.331 to 1.611)	<.001	.003
Change in perceived documentation time (minutes)	Median edit time (seconds)	0.006 (0 to 0.012)	0.006 (−0.001 to 0.012)	.06	.07
	Perceived ease of use (yes vs no)^c^	−4.144 (−6.156 to −2.132)	−4.149 (−6.149 to −2.15)	<.001	<.001

a IPW: inverse-probability-weighting based on a response propensity model.

b Coefficients are shown as β (95% CI). The unweighted model used all responders while the IPW model used a subset of the participants for which all necessary data were available (see Multimedia Appendix 7 for comparison and details).

c The reference group for "perceived ease of use" consisted of only approximately 15 respondents who rated the scribe as not easy to use; estimates from this small subgroup should be interpreted with caution, as the linear regression model is highly sensitive to outliers within such a small reference group, which might skew the magnitude of the reported coefficient.

## Discussion

### Principal Findings

Previous studies have demonstrated that AI medical scribes can reduce perceived documentation time as well as stress, thereby potentially lowering the risk of burnout among clinicians [15,16,29]. However, these findings have almost exclusively originated from the United States, whose health care system differs substantially from European systems in terms of organization, financing, and clinical workflows [12-16]. In our study, we showed that, in a European context, the use of an AI medical scribe was similarly associated with reduced physician-reported stress and a decrease in self-reported time spent on documentation.

Our findings on perceived time savings are consistent with the majority of published studies. However, not all studies have been able to demonstrate a reduction in documentation time [18]. In most prior work, time has been assessed subjectively, as in our study, but objective time measurements reported in some studies also support the observation that AI medical scribes can reduce the time burden of documentation [14,16]. However, due to inherent study design limitations, neither our study nor similar studies in this field can establish causality [29].

Previous studies have additionally reported differences between professional groups in the time spent in clinical documentation [7]. In contrast, we did not detect meaningful differences between professions in our analysis, suggesting that the potential advantages of AI medical scribe use may generalize across clinician groups in this setting.

Second, Liu et al [18] observed that the extent of clinicians’ use of an AI medical scribe influenced the magnitude of benefit they derived in their daily work. In contrast, in our study we did not observe any substantive change in editing time between the initial phase of use and subsequent periods. This may suggest that efficiency gains, at least with respect to note editing time, are attained relatively early, remain comparatively stable over time, and are indicative of a technology that is readily usable and intuitive without a substantial learning period. Alternative interpretations should, however, be acknowledged. Users completed approximately 5 hours of structured onboarding before the observation window began, so the flat trajectory is equally consistent with a ceiling effect in which most efficiency gains were realized during training prior to measurement. We also note that editing time alone may not capture other dimensions of learning such as improved note quality, reduced cognitive effort, or more effective use of advanced features, which could continue to evolve after editing time has stabilized.

In our study, the time required to edit notes generated by the AI medical scribe was relatively short, with a median of 93 seconds. This may partly reflect the structured onboarding provided to users (approximately 5 hours), which could have increased familiarity with the interface and editing workflow. Notably, a subset of editing episodes extended over several hours. Because editing time was defined as the interval between the first and last recorded edit, periods of inactivity were not separable from active editing. Prolonged intervals may therefore reflect a common workflow in which the editing window is left open and the note is finalized later, rather than sustained continuous editing. A significant number of notes were not edited at all (approximately 42% of all generated notes), and the fate of this group has important but opposing implications for interpretation. Onboarding follow-ups with users suggested at least three distinct patterns: (1) notes accepted as generated and transferred directly to the EHR, which would imply very high draft quality and any efficiency benefit captured in our primary analysis; (2) notes transferred to the EHR and then edited within the EHR rather than in the AI medical scribe interface, which would represent unmeasured documentation work and could overstate the observed efficiency gain; and (3) notes abandoned entirely and rewritten from scratch, which would imply overestimated usefulness. Because transfer status and posttransfer edits in the EHR were not available to the research team, we cannot quantify the relative prevalence of these patterns, and the direction of any net bias is therefore uncertain. Lukac et al [16] have recently reported a comparable inability to verify posttransfer edits in the EHR in a randomized trial of an ambient AI scribe and noted that this constraint limits inference about true documentation savings.

Both visit type (in-person, digital, and telephone) and editing platform (desktop vs mobile) were associated with variation in editing time. Remote encounters resulted in approximately 62% and 55% less editing time for digital and telephone contacts, respectively. On mobile platforms, editing times were roughly double those on the desktop platform. Editing on a desktop interface was also linked to greater editing efficiency. Although the workflow is essentially identical across platforms, we hypothesize that this difference reflects input modality constraints. Touch screen keyboards, limited screen real estate, and the lack of multiwindow navigation likely slow text editing, review of longer passages, and cross-referencing on mobile compared with desktop. Mobile use may also occur predominantly in less ergonomic contexts, such as between visits or at the bedside. Given that improved documentation efficiency is one of the main drivers for adopting AI medical scribes, these findings highlight the importance of careful selection and optimization of the user interface. Without attention to workflow design and platform choice, the full potential benefits of the technology may not be realized in routine clinical practice.

Interestingly, physicians’ self-reported documentation time was substantially longer than the measured note editing time. This suggests that, within the time they attribute to “documentation,” clinicians are also performing other tasks, such as writing prescriptions or referrals requiring navigation in the EHR. The estimated time is, however, broadly in line with a previous study [12]. Conversely, prior work indicates that although health care professionals systematically overestimate the time spent on documentation, often by as much as 2-fold [25], there remains a moderate correlation between actual and estimated time, supporting the use of clinician self-reports as a pragmatic surrogate measure in large-scale process improvement and research [26,27]. This recall bias is also a potential limitation in our study. In addition, the survey item asked clinicians how many minutes they spend finalizing a typical clinical note, while we interpret this self-reported time as encompassing the full documentation workflow (including navigation, prescriptions, and referrals). If respondents understood the question more narrowly, the estimates may under- or overstate the broader time impact, underscoring the need for additional metrics that capture the full scope of clinical documentation work. The significant number of notes excluded from the analysis due to no editing likely had varying fates such as used as is without editing, transferred and edited in the medical record system, or were disregarded.

One important dimension of effectiveness is health care professionals’ experience, which is particularly critical in the context of worsening shortages and increasing burnouts of medical personnel [30,31]. The urgency of addressing clinician well-being is also reflected at the policy level: for example, the National Academy of Medicine convened a meeting in December 2024 to examine the potential for AI to improve health worker well-being, including reducing burnout [32]. This growing international interest highlights how AI-based tools, such as AI medical scribes, are increasingly viewed not only as instruments for improving efficiency but also as potential levers to mitigate the psychological burden of clinical work.

In our study, the reduction in stress was associated both with the perceived ease of use of the AI medical scribe and with shorter editing time for the generated notes. Although these findings could partly be explained by simpler clinical cases requiring less editing, the existing literature does not support the point of view [13,33], and they may reflect a benefit attributable to automation, associated with a reduced self-assessed workload. This suggests that, beyond potential gains in efficiency, AI medical scribes may contribute meaningfully to improving clinicians’ day-to-day work experience, which is a key outcome in its own right. This interpretation is in line with previous studies reporting that AI medical scribes can reduce clinicians’ cognitive load and, consequently, alleviate stress and the risk of burnout [15].

### Strengths and Limitations

The main strength of this study is the large number of medical notes analyzed, which likely provides a reliable representation of current editing times in routine clinical practice. Additionally, we excluded the 138,196 notes with no recorded edits to avoid reporting overly positive or potentially misleading results. If a clinician did not use the generated note or modified it later within the EHR system, actions not captured in our dataset, retaining these notes could have artificially inflated efficiency estimates. Some of the excluded notes were likely used as generated, but we were unable to reliably distinguish these cases.

However, we were unable to determine whether the edits made by professionals were clinically necessary for medical quality or patient safety, or whether they primarily reflected stylistic preferences, tone adjustments, or other experiential aspects of documentation. Previous research has, however, demonstrated quite equal quality of doctor and AI-generated medical documentation [34]. The most important limitations relate to the study design. Although this was a mixed methods study, the survey component was not controlled: it was administered only once and did not include a comparison group. Although response propensity weighting suggested that nonresponse bias was unlikely to substantially influence the results, residual bias from unmeasured factors cannot be excluded. In addition, the survey sample consisted of users who had accumulated sufficient use of the AI medical scribe, specifically, fully onboarded clinicians who had used the tool for at least 3 months, generated more than 100 notes, and used the advanced conversational edit feature, which likely overrepresents professionals who were more satisfied or at least willing to continue using the tool. Users who discontinued use early or struggled with adoption were therefore likely excluded, introducing potential survivor bias and limiting the generalizability of the findings to the broader clinician population. Also, since the ease-of-use variable was highly skewed toward positive responses, its estimates should be interpreted cautiously as reflecting differences between a relatively small subgroup of respondents reporting lower usability. Because the reference group consisted of only approximately 15 respondents, the linear regression model is highly sensitive to outliers within this extremely small subgroup, which might skew the magnitude of the reported estimate. We were unable to reliably assess whether perceived time savings varied by clinicians’ prior documentation method (eg, typing, dictation, and human scribe), as several subgroups were too small to yield stable estimates, leaving potential heterogeneity across baseline workflows unresolved. Also, the survey responders were predominantly composed of general practitioners and, therefore, the survey results cannot readily be generalized across specialties based on these data. Furthermore, the primary survey item asked clinicians how many minutes they spend finalizing a typical clinical note, which is narrower than the broader documentation time construct discussed throughout this manuscript; respondents may not have considered tasks such as EHR navigation, prescription writing, or referral management when answering this prompt, and the magnitude of any time savings beyond note finalization therefore cannot be quantified from these data.

As in almost all prior work in this area, time savings could not be quantified fully objectively. Both self-reported documentation time and editing time are subject to multiple sources of error reported also in previous studies [16]. However, because a comparative baseline reflecting preimplementation workflow could be constructed only for the self-reported time measure, it was selected as the primary outcome variable. The editing time has several potential sources of error. While current software allows relatively precise estimation of time spent using the AI medical scribe (eg, editing time), no baseline time measurements of documentation without the tool were available. Editing time data are consistent with AI medical scribe technology being able to support efficient documentation, but it does not on its own provide an absolute estimate of time saved. Moreover, this study evaluated editing duration, self-reported user experience, and adoption patterns, but it did not assess note accuracy, error types, clinical quality, or patient safety outcomes; comprehensive evaluation of these dimensions would require further research, including systematic chart review, safety event monitoring, and assessment by downstream clinicians of the clinical usability of the generated notes. To more robustly assess effectiveness, future studies should incorporate objectively measured baseline and follow-up documentation times, include the full spectrum of users (including those who discontinue or are dissatisfied), and evaluate patient experience, ideally within a randomized controlled study design.

### Conclusions

Our findings suggest that the perceived time savings and reductions in cognitive load and stress reported in previous US-based studies may also be achievable within a European health care system using an AI medical scribe. These results reflect the experience of sustained, fully onboarded adopters mainly in general practice and may not generalize to the broader clinician population, particularly those who discontinued use or never fully adopted the tool. The single-arm observational design and reliance on retrospective self-report are important considerations when interpreting these associations.

## Supplementary material

10.2196/90052 Multimedia Appendix 1 Full survey instrument (translated from Swedish).

10.2196/90052 Multimedia Appendix 2 Proportion of professions included in the study. The professions in “other” consist of less than 2% of the total professionals per profession.

10.2196/90052 Multimedia Appendix 3 Comparison of respondents and nonrespondents.

10.2196/90052 Multimedia Appendix 4 Histogram with log-scaled axis.

10.2196/90052 Multimedia Appendix 5 The Likert shift distributions.

10.2196/90052 Multimedia Appendix 6 Descriptive analysis comparing characteristics of notes with versus with no edits.

10.2196/90052 Multimedia Appendix 7 Linear regression models (unweighted vs IPW-weighted) coefficients are shown as β (95% CI). IPW indicates inverse probability weighting based on a response propensity model.

## Abbreviations

AI: artificial intelligence
EHR: electronic health record
GDPR: General Data Protection Regulation
IPW: inverse-probability-weighted
SQUIRE: Standards for Quality Improvement Reporting Excellence
