# Supplementary figures and images for: Evaluation of an AI Medical Scribe After 236,153 Notes Generated Across Care Levels in a European Health System: Mixed Methods Retrospective Observational Study
Source: JMIR Med Inform. 2026 Jul 10;14:e90052. doi: 10.2196/90052 (PMC13354122; doi:10.2196/90052)

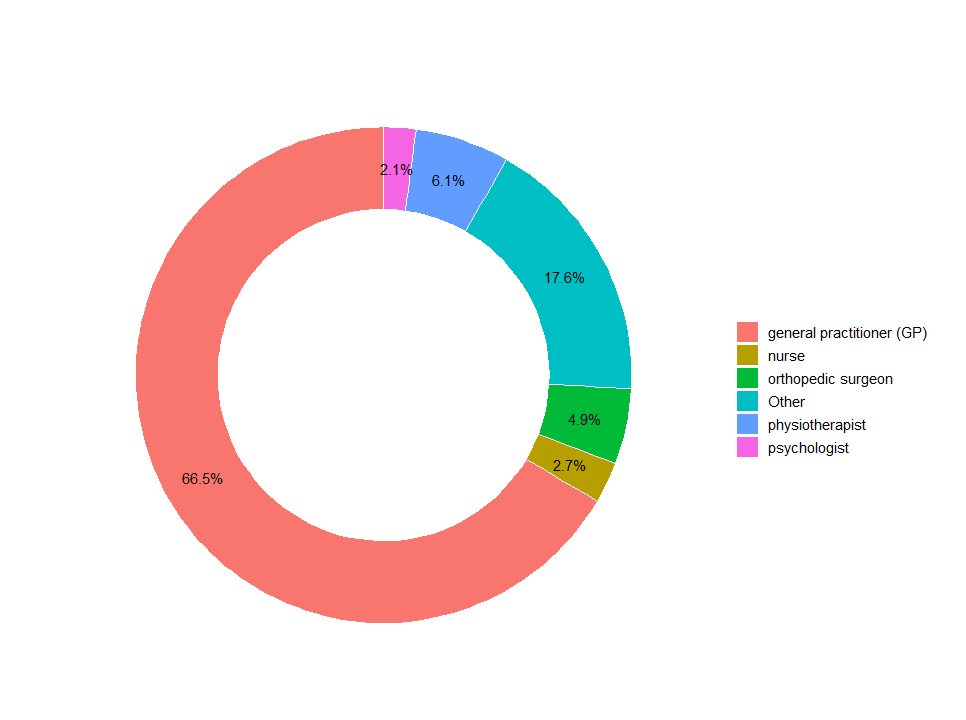

Supplement: Multimedia Appendix 2 [file medinform-v14-e90052-s002.docx]

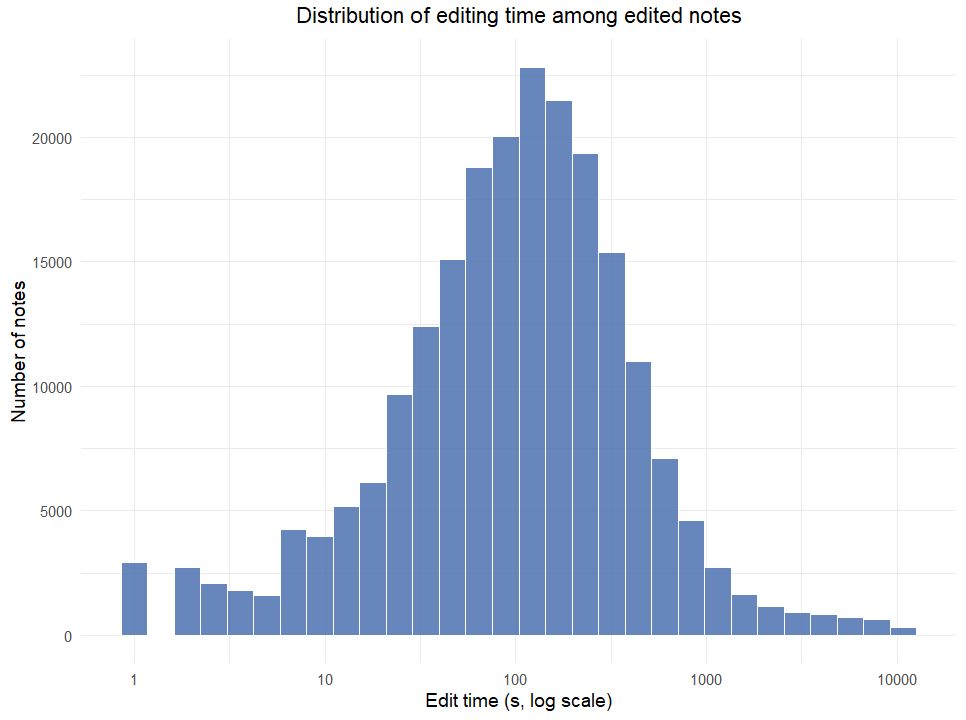

Supplement: Multimedia Appendix 4 [file medinform-v14-e90052-s004.docx]

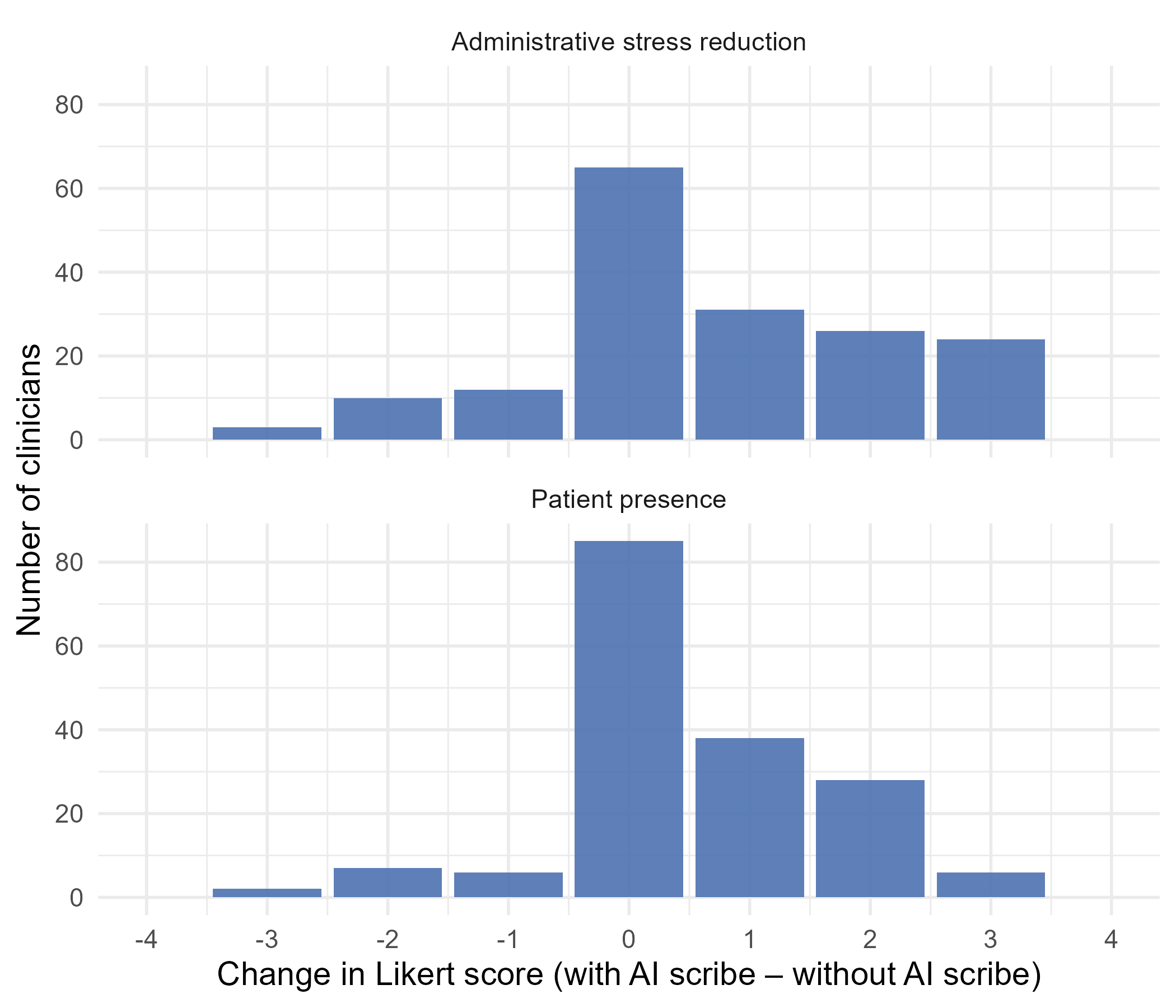

Supplement: Multimedia Appendix 5 [file medinform-v14-e90052-s005.docx]
