# Supplementary material for: Evaluation of an AI Medical Scribe After 236,153 Notes Generated Across Care Levels in a European Health System: Mixed Methods Retrospective Observational Study
Source: JMIR Med Inform. 2026 Jul 10;14:e90052. doi: 10.2196/90052 (PMC13354122; doi:10.2196/90052)
Supplement: Multimedia Appendix 3 [file medinform-v14-e90052-s003.docx]

| **Characteristic** | **Non-responders**  N = 322^1^ | **Responders**  N = 152^1^ | **p-value**^2^ |
| --- | --- | --- | --- |
| Clinic type |  |  | <0.001 |
| Primary care | 223 (69%) | 134 (88%) |  |
| Secondary/Hospital care | 99 (31%) | 18 (12%) |  |
| Profession |  |  | <0.001 |
| general practitioner (GP) | 241 (75%) | 146 (96%) |  |
| nurse | 2 (0.6%) | 1 (0.7%) |  |
| orthopedic surgeon | 23 (7.1%) | 0 (0%) |  |
| Other professions | 35 (11%) | 4 (2.6%) |  |
| physiotherapist | 14 (4.3%) | 1 (0.7%) |  |
| psychologist | 7 (2.2%) | 0 (0%) |  |
| Number of generated notes | 336 [210, 499] | 371 [217, 564] | 0.3 |
| Median edit time (s) | 96 [50, 172] | 88 [53, 168] | 0.4 |
| ^1^n (%); Median [interquartile range Q1, Q3] | | | |
| ^2^Pearson's Chi-squared test; Wilcoxon rank sum test | | | |
