# Supplementary material for: Evaluation of an AI Medical Scribe After 236,153 Notes Generated Across Care Levels in a European Health System: Mixed Methods Retrospective Observational Study
Source: JMIR Med Inform. 2026 Jul 10;14:e90052. doi: 10.2196/90052 (PMC13354122; doi:10.2196/90052)
Supplement: Multimedia Appendix 7 [file medinform-v14-e90052-s007.docx]

| Outcome | Predictor | Unweighted β  (95% CI) | IPW-weighted β  (95% CI) | Unweighted P value | IPW-weighted P value |
| --- | --- | --- | --- | --- | --- |
| Δ Stress | Median edit time (seconds) | -0.003  (-0.005, -0.001) | -0.003  (-0.006, -0.001) | 0.01250000 | 0.0090800 |
|  | Perceived ease of use (yes vs no) | 1.796  (1.043, 2.549) | 1.62  (0.85, 2.389) | 0.00000509 | 0.0000538 |
| Δ Presence | Median edit time (seconds) | -0.001  (-0.002, 0.001) | 0  (-0.002, 0.002) | 0.51800000 | 0.6900000 |
|  | Perceived ease of use (yes vs no) | 1.199  (0.578, 1.821) | 0.971  (0.331, 1.611) | 0.00019400 | 0.0031800 |
| Δ Recall | Median edit time (seconds) | 0.006  (0, 0.012) | 0.006  (-0.001, 0.012) | 0.05680000 | 0.0719000 |
|  | Perceived ease of use (yes vs no) | -4.144  (-6.156, -2.132) | -4.149  (-6.149, -2.15) | 0.00007280 | 0.0000673 |
