# Supplementary material for: Evaluation of an AI Medical Scribe After 236,153 Notes Generated Across Care Levels in a European Health System: Mixed Methods Retrospective Observational Study
Source: JMIR Med Inform. 2026 Jul 10;14:e90052. doi: 10.2196/90052 (PMC13354122; doi:10.2196/90052)
Supplement: Supplementary file 5 [file medinform-v14-e90052-s006.docx]

Characteristic	Edited<br>N = 236,153[1]	No edits recorde<br>N = 138,196[1]
Visit type		
Admin/other/unknown	44,832 (19.0%)	37,032 (26.8%)
Digital	3,235 (1.4%)	3,335 (2.4%)
Physical encounter	176,460 (74.7%)	85,716 (62.0%)
Telephone encounter	11,626 (4.9%)	12,113 (8.8%)
Interaction platform		
Desktop	215,643 (91.3%)	125,635 (90.9%
Mobile	2,396 (1.0%)	2,073 (1.5%)
Unknown	18,114 (7.7%)	10,488 (7.6%)
Clinic type		
Other	5 (<0.1%)	29 (<0.1%)
Primary care	182,780 (77.4%)	107,727 (78.0%
Secondary/Hospital care	53,368 (22.6%)	30,428 (22.0%)
Unknown	0 (0%)	12 (<0.1%)
Professions		
General practitioner (GP)	195,450 (82.8%)	111,610 (80.8%
Nurse	1,598 (0.7%)	2,695 (2.0%)
Orthopedic surgeon	9,233 (3.9%)	3,885 (2.8%)
Other professions	19,559 (8.3%)	15,524 (11.2%)
Physiotherapist	7,026 (3.0%)	2,437 (1.8%)
Psychologist	3,287 (1.4%)	2,045 (1.5%)
